# Metagenomic and paleopathological analyses of a historic documented collection explore ancient dental calculus as a diagnostic tool

**DOI:** 10.1038/s41598-024-64818-7

**Published:** 2024-06-26

**Authors:** Rita M. Austin, Tanvi P. Honap, Allison E. Mann, Alexander Hübner, Cassandra M. S. DeGaglia, Christina Warinner, Molly K. Zuckerman, Courtney A. Hofman

**Affiliations:** 1 Frontiers in Evolutionary Zoology Research Group, Natural History Museum of Oslo, University of Oslo, Oslo, 0562 Norway; 2 Department of Anthropology, National Museum of Natural History, Smithsonian Institution, Washington, DC 20560 USA; 3 Department of Anthropology, University of Oklahoma, Norman, OK 73019 USA; 4 Laboratories of Molecular Anthropology and Microbiome Research, University of Oklahoma, Norman, OK 73019 USA; 5 Department of Biological Sciences, Clemson University, Clemson, SC 29634 USA; 6 Department Archaeogenetics, Max-Planck-Institute for Evolutionary Anthropology, Leipzig, 04103 Germany; 7 Department of Anthropology, Tulane University, New Orleans, LA 70118 USA; 8 Department of Anthropology, Harvard University, Cambridge, MA 02138 USA; 9 Department of Anthropology and Middle Eastern Cultures, Mississippi State University, Mississippi State, MS 39762 USA

**Keywords:** Pathogen identification, Robert J. Terry Collection, Ancient DNA, Dental calculus, Interdisciplinary, Tuberculosis, Bacterial infection, Tuberculosis, Metagenomics, Skeleton

## Abstract

Dental calculus is a microbial biofilm that contains biomolecules from oral commensals and pathogens, including those potentially related to cause of death (CoD). To assess the utility of calculus as a diagnostically informative substrate, in conjunction with paleopathological analysis, calculus samples from 39 individuals in the Smithsonian Institution’s Robert J. Terry Collection with CoDs of either syphilis or tuberculosis were assessed via shotgun metagenomic sequencing for the presence of *Treponema pallidum* subsp*. pallidum* and *Mycobacterium tuberculosis* complex (MTBC) DNA. Paleopathological analysis revealed that frequencies of skeletal lesions associated with these diseases were partially inconsistent with diagnostic criteria. Although recovery of *T. p. pallidum* DNA from individuals with a syphilis CoD was elusive, MTBC DNA was identified in at least one individual with a tuberculosis CoD. The authenticity of MTBC DNA was confirmed using targeted quantitative PCR assays, MTBC genome enrichment, and in silico bioinformatic analyses; however, the lineage of the MTBC strain present could not be determined. Overall, our study highlights the utility of dental calculus for molecular detection of tuberculosis in the archaeological record and underscores the effect of museum preparation techniques and extensive handling on pathogen DNA preservation in skeletal collections.

## Introduction

The origins and evolutionary histories of acquired syphilis and tuberculosis (TB) are of great interest to researchers across the social, natural, and health sciences because of their significant social, demographic, and health impacts on human populations^1–8^. Historically referred to as the “Great Pox” and “consumption”, syphilis and TB, respectively, are communicable diseases with high worldwide morbidity and mortality rates^7,9–12^. The WHO estimates 7.5 million people were newly diagnosed with TB in 2022^13^ and 8 million people, between 15 and 49 years old, acquired syphilis in 2022^14^.

Syphilis is a sexually transmitted infection caused by the bacterium *Treponema pallidum* subsp. *pallidum*. Untreated syphilis is a multi-stage, chronic inflammatory disease that can systemically affect the body, including the cardiovascular, neurological, and skeletal systems^15,16^. Since the recognition of syphilis in Europe ~ 500 years ago, vigorous debate has revolved around its origins and antiquity^1,6,17,18^. Because syphilis (e.g., treponemal infection) can sometimes generate highly specific, diagnostic skeletal lesions, debate over its origins and antiquity, as well as subsequent investigations of disease burden in past populations, has regularly been grounded in skeletal evidence^1^. Genomic analysis of *T. p. pallidum* is notoriously difficult even for modern clinical samples^19^, in part due to the bacterium’s inability to live and reproduce outside of a host (e.g., humans, purposefully infected rabbits)^20,21^. Positive molecular identification of *T. p. pallidum* from skeletal material has also been extremely difficult^20,22^ and only recently achieved. Further molecular investigations of *Treponema* species and diseases typically follow the identification of skeletal lesions at least consistent with treponemal infection via paleopathological analyses. Molecular investigations typically proceed with direct sampling of specific individuals’ skeletal lesions^18,23^, teeth, or petrous portions, such as from individuals with skeletal evidence of congenital infection, which produces a very high bacterial load^23–27^.

TB is caused by bacteria belonging to the *Mycobacterium tuberculosis* complex (MTBC), including *M. tuberculosis *sensu stricto which causes the majority of human TB cases. While TB typically affects the lungs, any bodily system can be affected including the renal, neurological, and skeletal systems. Symptoms and disease severity depend on the host’s immune response, inoculum load, and the causal bacterial strain, with TB often being fatal without proper treatment^28,29^. As with syphilis, investigations into the evolution, antiquity, and past disease burden of TB have been anchored in skeletal evidence, as active TB infection can generate distinctive skeletal lesions^30–35^. Analysis of ancient MTBC DNA from human skeletal individuals from the pre-contact era Americas have helped elucidate the zoonotic transmission of MTBC strains between humans and non-human animals, such as pinnipeds, in antiquity^4,36^. While there is some debate regarding the timing of the origin of the MTBC^4,37–41^, this bacterial complex has likely co-evolved with human populations for at least several thousand years^4,37–40,42^.

Evolutionary understanding of how, when, and where syphilis and TB have affected human populations in the past relies upon their positive or probable identification from temporally and spatially contextualized human skeletal individuals^1,2^. Calcified dental plaque (dental calculus) has emerged as a reservoir of high-quality human, microbial (including pathogen), and dietary biomolecules^43–47^. Because dental calculus is a biofilm, in addition to its easy access and simple sampling procedures, its collection and testing is generally considered to be comparatively less destructive to human skeletal material than the typical use of dental pulp or bone powder from pathological lesions or the petrous portion for molecular analysis^48^. Due to the oral location of dental calculus, oral pathogens have been the primary focus of disease-oriented research. However, the well-documented relationship between oral and systemic health^49–52^ makes dental calculus an opportune substrate to investigate systemic pathogens as well. This is especially true for pathogens that do not commonly affect bone or dental tissues, or those that infrequently and inconsistently generate highly specific skeletal lesions. *T. p. pallidum* and the MTBC meet both criteria. Identification of pathogen biomolecules in dental calculus could thus make many skeletally “invisible” diseases identifiable and analyzable in the past^53,54^. However, broader understanding of dental calculus as a diagnostic tool remains necessary.

The current study explores the recoverability of *T. p. pallidum* and MTBC DNA^55^ from dental calculus using shotgun metagenomic sequencing and targeted, quantitative PCR and whole-genome capture methods, alongside osteological and paleopathological assessment of individuals from the Smithsonian Institution’s (SI) Robert J. Terry Anatomical Collection (Terry Collection). Here, we combine molecular and macroscopic paleopathological approaches and synthesize these datasets to explore the potential of dental calculus as a diagnostic tool. Systemic proliferation of *T. p. pallidum* during early stage (primary, secondary) syphilis, and *M. tuberculosis* within pulmonary and systemic TB infections make these pathogens ideal for testing the diagnostic potential of past human infectious diseases using dental calculus^15^. Additionally, the Terry Collection contains individuals with antemortem, clinical diagnoses (cause of death; CoD) of syphilis and TB, allowing us to verify our ability to detect and diagnose the presence of infectious diseases within dental calculus from the past (e.g., historical collections, bioarchaeological and biohistorical contexts). To our knowledge, this is one of the first^56^ investigations of dental calculus as a potential reservoir of MTBC and *T. p. pallidum* DNA.

## Results

### Shotgun metagenomic sequencing

**Figure 1:**
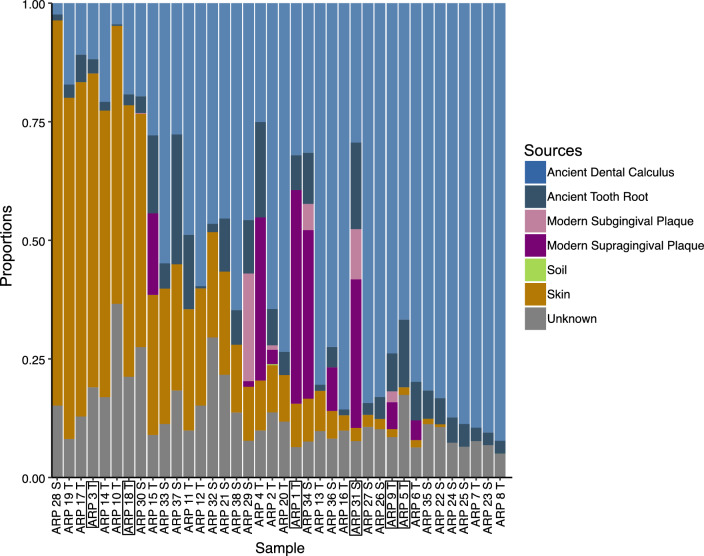
SourceTracker analysis of 16S rRNA metagenomic reads sorted by increasing proportions of taxa associated with archaeological dental calculus samples^58^. Boxed sample labels indicate samples used in hybridization capture experiments.

An average of 16.8 million reads (range = 6.2—96 million) were recovered across all samples, with extraction and library blanks sequenced to an average of 3 million reads. SourceTracker^57^ analysis revealed recognizable oral signatures for all samples (Fig. 1 and Supplementary Fig. S1). Consistent with the collection’s lack of burial history, microbes from soil environments were recovered in negligible amounts. The proportion of skin-associated microbes varied across samples, potentially due to the long-term historical curation of the Terry Collection; individuals have been extensively handled without gloves, long before biomolecular technologies became possible applications. While dental calculus samples with high proportions of skin-associated microbes may not be suitable for analyses focusing on overall community structure and ecology, they remain suitable for investigating specific pathogens.

Initial assessment of the shotgun metagenomic data using Heuristic Operations for Pathogen Screening (HOPS)^59^ showed several samples passing damage pattern and edit distance filters for genus *Treponema* (see red boxes in Supplementary Fig. S2). However, upon further inspection, this screening only identified *T. putidum* and *T. denticola* with greater than 80% certainty across samples for *“Treponema”*. Across all samples, HOPS results also showed little to no reads mapping to any MTBC species.

Using a reference-based mapping approach, 19 samples had > 1000 unique reads mapping to the ancestral MTBC reference genome (Figs. S3 and S4, and Supplementary Table S2), as opposed to none for *T. p. pallidum* (Supplementary Figs. S5 and S6, and Supplementary Table S3). Overall, reads mapping to the MTBC genome showed higher breadth of coverage and lower clonality (2.7–22.5 mapped reads/unique q37 reads) in contrast to reads mapping to *T. p. pallidum,* which had low breadth of coverage, high clonality (35.1–861.7 mapped reads/unique q37 reads), and “stacking” of reads in conserved regions of the genome, suggestive of non-specific mapping. Illustrating differential recovery of oral bacteria, all samples had higher mapping rates to the ancestral MTBC reference genome (0.0008–0.0278%), as well as to the reference genomes of common oral bacteria, *Fusobacterium nucleatum* (0.00005–0.08914%) and known oral pathogens *Porphyromonas gingivalis* (0.00001–0.36322%) and *Tannerella forsythia* (0.00009–9.29344%), than to *T. p. pallidum* (0.00001–0.00072%) (Supplementary Fig. S7). A Kruskal–Wallis rank sum test (KW chi-square value = 86.9, *P* =  < 2.2e− 16) found significant differences between the proportion of reads mapping among reference genomes (Supplementary Table S4). Pairwise Wilcoxon tests of mapping rates among reference genomes revealed significant differences (*P* =  < 0.05) between all reference genomes, except between *F. nucleatum* and *P. gingivalis;* and *F. nucleatum* and *M. tuberculosis* (Supplementary Table S5). With < 0.007% mapping rates, *T. p. pallidum* DNA was functionally undetectable across all samples. Therefore, further analyses on *T. p. pallidum* were not conducted.

### MTBC quantitative PCR screening

None of the DNA extracts tested positive for the rpoB2 assay^60^ targeting the mycobacterial *rpoB* gene (Supplementary Table S6). Only ARP 1 T, ARP 9 T, and ARP 18 T were positive for the IS6110 assay^60^ targeting the MTBC-specific multi-copy insertion element IS6110 (Supplementary Table S7). *M. tuberculosis* H37Rv DNA and DNA extracts from the Peruvian mummies used as positive controls (samples 54U, 58U, and 64U)^60^ tested positive for both qPCR assays. Extraction controls tested negative, confirming the validity of the assays.

### MTBC capture

Our in-solution MTBC whole-genome capture used RNA baits generated from modern *M. tuberculosis* strains. It was successful in reconstructing MTBC genomes from bone samples 54U and 64U from Peruvian mummies, which previously yielded nearly complete MTBC genomes^4^. Specifically, for samples 54U and 64U, we recovered up to 96% and 73% of the MTBC genome with an average coverage of 39- and 2.3-fold, respectively (Supplementary Table S8). The implemented bait set was markedly different from those previously applied for MTBC genome enrichments^4,36^, which have typically used synthetic baits spanning the entire MTBC genome. While generating synthetic baits allows the exclusion of unwanted genomic regions, such as repeat elements, the RNA bait set is significantly less expensive, further increasing the appeal of this approach^61^.

For the six ARP samples that were enriched for the MTBC genome, < 0.01% reads mapped to the ancestral MTBC reference genome^55^ across all samples. The percentage of the MTBC genome recovered ranged from 0.03 to 12.45% (Supplementary Table S8). High cluster factors across samples indicates all reads were likely sequenced. In addition to the positive controls (Peruvian mummy samples 54U and 64U), only ARP 1 T had > 1000 unique mapped reads following BLASTN filtering, which we considered to be authentic MTBC reads. These reads showed damage patterns as expected for ancient DNA (Supplementary Fig. S8). Samples 54U and 64U had, respectively, 930 and 104 homozygous SNPs identified; the fewer number of SNPs in 64U as compared to findings from Bos et al.^4^ is due to the lack of sufficient coverage obtained for this sample in this study. Only 11 homozygous SNPs were identified in ARP 1 T (Supplementary Table S9). Sample 54U was identified to belong to the *M. pinnipedii* lineage, confirming previous results^4^; however, MTBC lineages could not be identified for samples 64U and ARP 1 T.

### MTBC authentication simulations

In silico simulations support robust identification of MTBC DNA from shotgun and hybridization capture data. While only a small proportion of total simulated reads mapped to the ancestral MTBC reference genome across all fragment length and spike-in level datasets (100bp: 9.04% ± 0.14%; 75bp: 6.87% ± 0.08%; 50bp: 4.45% ± 0.16%; 30bp: 2.97% ± 0.08%), very few mapped to a different *Mycobacterium* species (n = 4 reads) and only did so when reads were very short (30bp). Moreover, very few bacterial reads mapped to the ancestral MTBC reference genome (n = 8 reads), with all originating from a single species in our mock oral community dataset (*Neisseria elongata*) (Table 1).

**Table 1:** In silico simulations demonstrate high fidelity of simulated mapped reads to the ancestral MTBC reference genome.

Fragment length (bp)	spike-in level (%)	# of reads mapped to TB	Proportion of reads mapped to TB (%)	# of bacteria reads mapped TB	# of TB reads mapped to other *Mycobacterium*
100	0.1	1840	9.20	0	0
100	0.5	8971	8.97	0	0
100	1.0	17,882	8.94	0	0
75	0.1	1391	6.96	0	0
75	0.5	6867	6.87	1	0
75	1.0	13,602	6.80	1	0
50	0.1	853	4.27	0	0
50	0.5	4545	4.55	0	0
50	1.0	9093	4.55	0	0
30	0.1	609	3.05	3	0
30	0.5	2972	2.97	1	1
30	1.0	5764	2.88	2	3

The proportion of mapped reads across all spike-in levels of authentic MTBC DNA was consistent within datasets of the same length profile. Very few reads mapped to the wrong *Mycobacterium* species and only at low read length profiles (30bp). Additionally, few reads originating from a bacterial genome mapped erroneously to the ancestral MTBC reference genome suggesting that the results reported here are not the consequence of misassigned or mismapped reads.

### Skeletal inventories and lesion analysis

**Figure 2:**
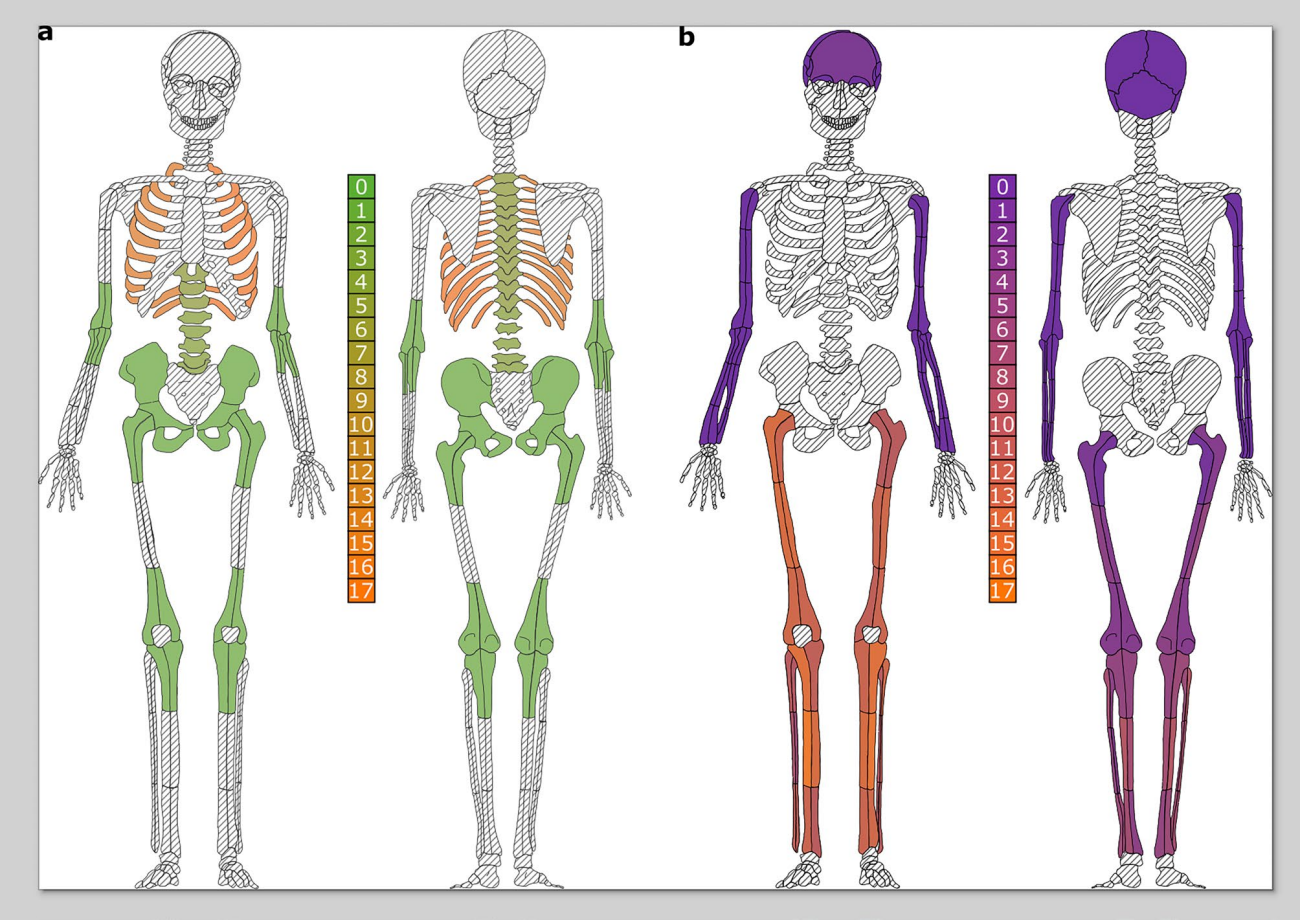
Frequency distributions of pathological skeletal lesions expressed as color gradients for skeletal elements across all 39 individuals, regardless of their recorded CoD. Frequency distributions (numbers) with color gradients (green and purple being less frequent than orange) of disease-indicating lesions are mapped onto an anatomical skeletal individual (modified from Buikstra and Ubelaker^62^). (**a**) Frequency distributions of lesions associated with TB (Supplementary Table S1, diagnostic criteria for TB); (**b**) Frequency distributions of lesions associated with syphilis/treponemal infection (Supplementary Table S1, diagnostic criteria for syphilis). Crosshatching indicates skeletal elements that were not assessed for disease-indicating lesions.

Syphilis/treponemal composite diagnostic scores ranged from 0 (lesions consistent with a non-treponemal disease process) to 5 (lesions specific to treponemal disease found on multiple skeletal elements or in the presence of lesions suggestive of treponemal disease on other skeletal elements). TB composite diagnostic scores ranged from 0 (lesions consistent with a non-tuberculosis process) to 3 (periosteal reactions—new sub-periosteal bone formation—on visceral surfaces of multiple ribs, and predominantly destructive, lytic lesions observed on more than one thoracic and/or lumbar vertebrae) (Supplementary Table S1). The frequency and distribution of lesions consistent with, suggestive of, and specific to syphilis/treponemal infection and those consistent and associated with TB across individuals are shown in Fig. 2. Composite diagnostic scores for the individuals are reported in Supplementary Table S10.

Because *T. p. pallidum* was functionally undetectable using shotgun metagenomic sequencing, percent of reads mapping to *T. p. pallidum* relative to composite diagnostic score could not be assessed for syphilis. No significant associations were detected between the percent of metagenomic reads mapping to MTBC and between the frequency and type of lesions associated with TB; composite diagnostic scores; or the six high diagnostic value indicators evaluated for TB^32^. However, a significant number of individuals (95%; *P* =< 0.0001) with a CoD of TB had lesions associated with TB (composite diagnostic score: ≥ 1), as did a smaller proportion (78%; *P* = 0.0005) of individuals with a CoD of syphilis (Supplementary Figs. S4 and S6). A small number of individuals with a syphilis CoD showed lesions suggestive of syphilis (31%; *P* = 0.0208) on one (10%; *P* =< 0.0001) (composite diagnostic score: 2) or more (21%; *P* = 0.0004) (composite diagnostic score: 3) skeletal elements. None of the individuals with a syphilis CoD had lesions specific to the condition. Similarly, 75% (*P* = 0.0006) of individuals with a CoD of TB had lesions associated with TB on either their ribs or vertebrae (composite diagnostic score: 1–2), while just 20% (*P* = 0.0002) manifest lesions on multiple ribs and vertebrae (composite diagnostic score: 3). Lastly, most individuals with a TB CoD (80%; *P* = 0.0002) have at least one high diagnostic value indicator, of which 75% were rib lesions (RIB2) (*P* = 0.0018); just 10% have more than one indicator (*P* =< 0.0001), all of which were vertebral lesions (BOD).

## Discussion

We investigated the potential of dental calculus as a diagnostic tool for identifying TB and syphilis infections in skeletal individuals. We integrated shotgun metagenomics and hybridization capture sequencing approaches with paleopathological assessments of individuals in the Terry Collection. Results varied across approaches and for each CoD, likely due to the complexity of disease presentation (see Supplementary Table S1 and Supplementary Figures S4 and S6). While most individuals with either TB or syphilis CoD expressed lesions associated with these conditions (see Supplementary methods), no associations could be made using shotgun metagenomic sequencing. Hybridization capture successfully recovered MTBC DNA, though only one individual, ARP 1 T, had sufficient reads to support positive MTBC identification. The limited molecular results are likely a result of the curatorial, preservation, and research history of the Terry Collection, as well as individual-specific dental calculus formation rates and bacterial disease pathogenesis.

Almost all individuals with a TB CoD showed lesions strongly associated with TB (i.e., composite diagnostic score = 1–2). Despite this, no substantial associations were found between MTBC DNA identified using shotgun metagenomic sequencing and TB-associated skeletal lesions (i.e., composite diagnostic score, high diagnostic value indicators, lesion frequency and distribution) for individuals with a TB CoD. However, this is likely an artifact of small sample size. Notably, the observed skeletal lesions strongly associated with TB primarily consisted of rib lesions (i.e., periosteal reactions on visceral rib surfaces, RIB2). Just 10% of individuals had vertebral lesions (i.e., destructive, lytic lesions on vertebrae; VEN2), and always with accompanying rib lesions. While not all living individuals with active TB infection will develop spinal lesions, spinal lesions often occur during later stages of the disease^63^. This is notable because, with the exception of four individuals, most individuals with a TB CoD are noted to have endured TB (i.e., active infection) for longer than one year (Supplemental Table S10). This would have allowed substantial time for the disease to progress. With the implementation of antibiotics against TB only beginning in 1945 (e.g., streptomycin), individuals assessed here, all of whom died before 1941, could have only been treated surgically or referred to sanitoria for extended supportive care^64^. Further, because individuals in the Terry Collection were generally of low socio-economic status (SES) to poor, with occupations that included “laborer”, “housework/wife”, “porter”, “poultry shop”, “photographer”, and “unknown”, their access to surgery and extended sanitoria treatments may have been economically and temporally constrained.

The small sample size precludes the use of an epidemiologic approach and limits generalizable interpretations. However, the combination of the scarcity of observable vertebral lesions in individuals with documented TB infection—most of several years duration—the molecular detection of *M. tuberculosis*, and the high prevalence of rib lesions, may justify some reconsideration of the de-prioritization of rib lesions as indicators of TB in paleopathological diagnostic criteria in some contexts^31,65,66^. At least in this sample of individuals, the diagnostic value of rib lesions is clearer; the very high prevalence of rib lesions, especially periosteal reactions (e.g., RIB1), across many individuals with a documented TB CoD and who experienced chronic TB, reinforces the diagnostic value and strong associations of rib lesions with TB^31,66,67^. As in all investigations, data collection and analysis is dependent on the availability and preservation of observable elements, as well as the synthesis of various approaches and diagnostic criteria to make determinations. However, the high degree of skeletal element representation and cortical bone preservation strengthen the interpretations made here, compared to less intact collections of skeletal individuals.

In juxtaposition to TB lesion observations, very few individuals with a syphilis CoD showed lesions associated with the condition. Further, most (84%) of the observed lesions were periosteal reactions, which have very low specificity to syphilis/treponemal infection^1,17,30,31,68^. This is because periosteal reactions are caused by a diversity of conditions and physiological and immunological processes^68–72^. Consistent with trends in paleoepidemiological analyses of syphilis/treponemal infections and the recognized low specificity and sensitivity of treponemal infection skeletal lesions^1,17^, few individuals (31%) showed lesions with higher diagnostic value (i.e., suggestive lesions). No one had lesions specific to the disease. Interpretive limitations due to small sample size are also exacerbated by the non-recovery of *T. p. pallidum*, using HOPS and reference-based mapping approaches, from these individuals’ dental calculus. Lacking any meaningful recovery of *T. p. pallidum* DNA, comparisons between molecular and skeletal data could not be made.

Of note, however, are the high frequencies of consistent (i.e., periosteal reactions) and moderately high frequencies of suggestive lesions amongst those with a syphilis CoD, mostly of tertiary stage (see Supplementary Table S10). This contrasts dramatically with expected frequencies; only 1–20% of untreated individuals living with syphilis are expected to experience any skeletal involvement. Within this, unknown but likely lower frequencies of distinctive, suggestive, and specific lesions are expected^73^. Higher instances of observed periosteal reactions in the present study cannot be easily attributed to sample bias towards pathological cases in the collection (see Supplementary Materials). Rather, they may reflect the life histories and biosocial lived conditions of individuals in the Terry Collection^74,75^. Established high rates of undernutrition and experiences of episodic to chronic physiological stress throughout the life course, coupled with high frequencies of local and systemic co-infection (e.g., TB, periodontal disease) and comorbidity (e.g., chronic and degenerative diseases) amongst individuals in the Terry Collection may have left them more vulnerable to hyperinflammatory responses to treponemal infection. In turn, these hyperinflammatory responses may have translated into higher frequencies of skeletal involvement than observed in less physiologically vulnerable individuals^75–77^. The higher instances may also be consistent with clinical reports of the underestimation of skeletal involvement in syphilis^78^. Alongside not recovering *T. p. pallidum* from dental calculus, as well as the established organismal fragility in scientific investigations, reliable identification of syphilis within skeletal material (excepting congenital cases) from archaeological or documented contexts remains exceptional.

Analysis of shotgun metagenomic data using a comparative screening approach, such as that implemented in HOPS, did not identify *T. p. pallidum* or MTBC reads in any individuals. Using a reference-based mapping approach, negligible numbers of *T. p. pallidum* reads were identified across all individuals. Combined with high clonality and stacking of reads in conserved regions of the genome, this suggests that these reads are non-specifically mapped. While several samples showed reads mapping to the ancestral MTBC reference genome, with relatively less clonality and read stacking, the amount of endogenous pathogen DNA does not correspond to CoD. The juxtaposition between the molecular identification of MTBC or *T. p. pallidum* and CoD information is highlighted by an individual who has a syphilis CoD but no CoD of TB, having the highest percentage of reads mapping to the ancestral MTBC reference genome (Supplementary Figures S4 and S6). Indeed, the co-infections documented within CoD designations do not show corresponding recovery of pathogen DNA for either disease. Although we were able to detect potential MTBC DNA, diagnosing individuals with either TB or syphilis using shotgun metagenomic sequencing of dental calculus alone was not possible.

Our study underscores the utility of targeted assays and sequencing for authentication of ancient MTBC DNA. Highlighting the utility of this assay as a preliminary screening tool for detection of TB in archaeological dental calculus, the MTBC-specific IS6110 insertion element was detected in three individuals with a TB CoD. However, MTBC qPCR assays may sometimes detect DNA from closely related mycobacteria such as *M. kansasii* or *M. smegmatis*, leading to false positive results^60^. Thus, confirmation of MTBC DNA using sequencing and authentication of ancient origin of those sequences by assessment of DNA damage patterns is necessary.

Our MTBC-genome capture, which used a cost-effective RNA bait approach as opposed to more expensive, commercially available synthetic probes, resulted in recovery of MTBC DNA from one of the three samples positive for the IS6110 qPCR assay, i.e., ARP 1 T. The hybridization capture resulted in a two-fold enrichment in endogenous MTBC DNA, with approximately 12% of the MTBC genome recovered post-capture, as opposed to only 0.42% pre-capture. Though not exhaustive of all mis-mapping that may happen between MTBC and other closely related *Mycobacterium* species, stringent post-mapping filtering using a BLASTN approach and authentication of DNA damage patterns, alongside supporting in silico simulations, suggest that the reads we recovered are authentic to MTBC. Although, the enrichment did not yield sufficient coverage to allow lineage determination of the MTBC strain, MTBC genome reconstruction and lineage assignment was successful for a positive control from a pre-contact Peruvian mummy^4^. This highlights the presented capture approach as an affordable and validated option for other researchers to use. Lastly, while a previous study has detected MTBC DNA in historic dental calculus using a qPCR assay^56^, this is, to the best of our knowledge, the first successful recovery of authenticated ancient MTBC DNA from dental calculus using high-throughput DNA sequencing.

Shotgun metagenomic analysis revealed that oral microbiome signatures were successfully preserved across most dental calculus samples. This shows the accessibility of microbial community and broader systemic health information from dental calculus. This is particularly meaningful because of the extremely short DNA fragments recovered across all individuals (between 31 and 74 bp). This is likely a direct result of the skeletonization/preservation process that was implemented for the Terry Collection, as well as the long history of intensive research conducted on individuals (see Supplemental Materials).

Much of the presented molecular results may be collection specific: the Terry Collection’s preservation and curation, especially its long history of extensive handling by thousands of researchers over many decades, have impacted molecular diagenesis, including the very short sequences (~ 31–74 bp) that were recovered. For example, in many historical documented collections, dental calculus was intentionally removed during collection preparation^79,80^. The handling of skeletal individuals also risks accidental removal^81^. In contrast, studies utilizing archaeological skeletal individuals and traditional sample types^18,23–27,36–40,42^ (e.g., dental pulp, bone powder) while not without challenges (e.g., contamination), may not be as hindered by the molecular diagenesis likely introduced by preparation, curation, and research history characteristic of historical documented collections like the Terry Collection, which may contribute to these studies’ success in pathogen detection.

Future research with other historical documented collections and/or recent-historical individuals, who endured different postmortem collection preparation procedures, and less subsequent handling, yet also have documented medical histories and clinical, antemortem diagnoses, as well as higher retained quantities of dental calculus, could yield more and longer pathogen DNA sequences. Systemic pathogen load, coupled with individual-specific rates of tartar buildup and dental calculus calcification, could also impact the likelihood of incorporation of pathogens into dental calculus. Specifically, high pathogen load and high rates of tartar deposition and calcification may increase incorporation and vice versa. Because TB and syphilis are acquired diseases, it is also possible that by sampling intact dental calculus (i.e., undisturbed or removed) more pathogen-specific DNA could be recovered. This is because as more recent layers of dental calculus deposition following infection and during active disease phases may contain more pathogen DNA. For example, Austin et al.^53^ recovered genomes of acute pneumonia-causing pathogens (*Klebsiella* *pneumonia* and *Acinetobacter* sp.) from dental calculus of a young man housed in the Terry Collection who had periodontal disease and oral abscesses. Similarly, Giffin et al.^82^ suggest that haematogenous permeability of pathogens into tissues is possible during sepsis-induced increased vascularity; as a byproduct, increased vascularity due to sepsis, could also increase pathogen incorporation into dental calculus. Sampling larger quantities of dental calculus and from teeth affected by abscesses or periodontal disease, both conditions that increase vascularity of dental tissues, may help optimize pathogen recovery from dental calculus^53^. However, careful consideration of sampling location and collection contextual information, alongside assessment of potential co-infections that can result in sepsis should also be considered in future studies. These considerations may be helpful for increasing the likelihood of recovering and eventually identifying more systemic infectious pathogens from skeletal individuals in the past, especially *Treponema*. Furthermore, a recent comparison of capture methods^83^ indicates that future studies using dental calculus may benefit from optimization of capture sequencing technology.

## Summary

Our findings have important implications for understanding ancient pathogen DNA recoverability and the utility of dental calculus for molecular diagnosis of TB and syphilis within human skeletal individuals. While *T. p. pallidum* DNA was not detected using shotgun metagenomics, MTBC DNA was detected in dental calculus using shotgun metagenomics and quantitative PCR methods, and authenticated using in-solution hybridization capture. In silico simulations suggest that the MTBC reads recovered from dental calculus are authentic and reliable. This, and the successful oral microbiome reconstructions presented here, indicate that dental calculus can yield broader systemic health information, beyond oral conditions. Pathogen DNA sequences recovered from shotgun metagenomics were not associated with individuals’ documented CoD in this study and are therefore inappropriate for extrapolating molecular diagnostic criteria. In contrast, the frequency and distribution of skeletal pathological lesions associated with TB across the skeleton were consistent with previous research. Syphilis/treponemal infection lesions, on the other hand, were observed more frequently than expected. Overall, while epidemiological generalizations are limited due to small sample size, the paleopathological results suggest that paleopathological diagnostic criteria for both diseases would benefit from reassessment. Further, the specific biomolecular results presented here may stem from the intense skeletal preparation methods (e.g., maceration) and decades of subsequent research on individuals in the Terry Collection. These findings emphasize the need for better understanding of differential biomolecular recovery across museum, historical, and archaeological skeletal collections^79^, especially for more careful consideration of destructive sampling of individuals from historical and archaeological contexts.

## Materials and methods

Based on contemporary policies, the Smithsonian Institution’s National Museum of Natural History Anthropology Department provided project and sampling approval. Further details regarding all analyses are given in the supplementary materials.

### Assessed individuals

Individuals (n = 39) in the Terry Collection were included in the present study based on documented antemortem CoD of either TB (n = 20) or syphilis (n = 19). A brief history and explanation of this study, the Terry Collection, and contributing individuals is provided in the supplementary material (see also Austin et al.^53^ and Zuckerman et al.^74^). Of note is our use of study-specific individual identifiers under the acronym Anonymous Research Participant (ARP). The de-identification applied here *vs.* the collection accession numbers assigned to these individuals was done to maintain these individuals’ genetic privacy (see Supplemental Materials). It was also done as a posthumous mechanism of recognition of these skeletal individuals as research participants. We also recognize the once-living individuals they represent *vs.* their scientific objectification and dehumanization during collection preparation, curation, and subsequent research^53,76,84^, though, we also recognize that ethical and respectful treatment of deceased individuals is cross-culturally variable^85^. Acknowledging skeletal individuals as interlocutors in research, both case study-based and population-level, follows a growing ethical precedent in biological anthropology^53,81,86,87^. We pair this with explicit acknowledgement that most individuals incorporated into the Terry Collection before the mid-twentieth century did not consent to anatomical dissection and subsequent incorporation into the Terry Collection, nor any of the subsequent research performed on this collection^53,74,88^. We also acknowledge that efforts to reinstate respect for people in the Terry Collection or individually thanking skeletal individuals does not represent restorative justice. They are also not solutions to the ethical issues surrounding the formation and subsequent research of historical documented collections such as the Terry Collection, nor the structural violence that these individuals and their communities endured before and after their death^53,74,85,88^. Rather, our actions are meant to be a mechanism for recognizing the humanity of these individuals and their roles as research contributors.

### Dental calculus DNA extraction and library preparation

DNA was extracted and prepared for sequencing at the Laboratories of Molecular Anthropology and Microbiome Research (LMAMR) in Norman, Oklahoma, USA, in a dedicated ancient DNA laboratory. Following previously published protocols^58^, between 1 and 5.5 mg of dental calculus was used for DNA extraction using a modified silica-column based purification method. Extracts were built into dual-indexed, double-stranded DNA libraries after a partial uracil-DNA-glycosylase (UDG) treatment^89^. Extraction and library negative controls were processed in parallel to monitor for contamination. Please see the Supplementary Materials for further information.

### Shotgun metagenomic sequencing and analysis

All libraries were shotgun-sequenced on an Illumina NextSeq machine (either 1 × 75 or 2 × 150) at the Max Planck Institute for the Science of Human History in Jena, Germany. Sequencing adapters were removed, and paired-end reads were merged and quality-filtered (minimum quality 20 and read length 25) using AdapterRemoval v2^90^ to generate analysis-ready reads. Successful retrieval of oral microbiomes was assessed using SourceTracker v1^57^. A genus-level taxonomic inventory based on the reads covering the 16S rRNA genes was determined for all libraries and used to assess the contributions of taxa from known “sources” such as oral, fecal, soil, and skin microbial communities. Analysis-ready reads were also subjected to the pathogen screening tool, HOPS^59^ for an additional assessment of pathogen profiles. To assess the recovery of DNA from common oral bacteria alongside *T. p. pallidum* and MTBC, analysis-ready reads were mapped to genomes of *T. p. pallidum* (GCA 000,604,125.1) and the computationally constructed ancestral MTBC reference genome^55^, as well as *Fusobacterium nucleatum* (GCA 000,007,325.1), *Porphyromonas gingivalis* (GCA 000,010,505.1), and *Tannerella forsythia* (GCA 000,238,215.1) using the Burrows-Wheeler Aligner (BWA) v0.7.12^91^ and parameters suited for ancient DNA. The breadth and depth of coverage for the three individuals with the most reads mapping to *T. p. pallidum* and the ancestral MTBC reference genome ^55^ were visualized using the BLAST Ring Image Generator (BRIG) program^92^. Statistical comparisons and visualizations were conducted in R^93^. See Supplementary Materials for further explanation.

### Targeted screening for MTBC DNA

#### Quantitative PCR assays

DNA extracts for all individuals’ with a tuberculosis CoD, and one individual with a syphilis CoD who showed a high number of MTBC mapped reads in the shotgun metagenomic data, were screened for the presence of MTBC DNA using quantitative PCR (qPCR, Roche Lightcycler 480) assays. Targeting the mycobacterial RNA polymerase B (*rpoB*) gene and the IS6110 multi-copy insertion element specific to the MTBC, assays followed previously established protocol^60^. Ancient DNA extracts from Peruvian mummy bone samples, which had previously yielded nearly complete MTBC genomes (samples 54U, 58U, and 64U)^60^, as well as DNA from the modern *M. tuberculosis* strain H37Rv, were included as positive controls.

#### MTBC genome capture and analysis

Six libraries were selected for whole-genome capture of MTBC DNA based on the percentage of unique, quality-filtered shotgun metagenomic reads mapped to the ancestral MTBC reference genome^55^ and the identification of MTBC-specific *rpoB* and/or IS6110 elements using qPCR assays. SourceTracker profiles of these samples confirmed that the majority of reads were attributed to taxa found in oral sources. A custom myBaits Whole Genome Enrichment kit (Arbor Biosciences), comprising biotinylated RNA baits that covered the entire *M. tuberculosis* genome, was used for the enrichment. Two rounds of hybridization capture were conducted following manufacturer’s instructions with slight modifications (see Supplementary Materials). Amplified enriched libraries were sequenced on an Illumina NextSeq (2 × 150 bp) run at the Oklahoma Medical Research Foundation Clinical Genomics Center in Oklahoma City, Oklahoma.

The resulting sequence data were processed using similar parameters as those used for shotgun metagenomic data analyses and mapped to the ancestral MTBC reference genome^55^. To authenticate that mapped reads originated from MTBC as opposed to from closely related non-MTBC mycobacteria, unique mapped reads were used as queries in a BLASTN^94^ search against the complete NCBI-nt database. Reads with non-MTBC matches among the top five results were removed. Variant calling was conducted using SAMTools *mpileup*^95^ and VarScan^96^ v2. MTBC lineages were determined with the SNP-IT tool^97^.

### MTBC authentication simulations

Because of the environmental ubiquity and close phylogenetic relationships within the genus *Mycobacterium,* in conjunction with the short fragment lengths inherent to ancient DNA, in silico simulations were conducted to assess rates of mis-mapping due to relative abundance of *Mycobacterium* and various read lengths. Simulated ancient DNA datasets from the ancestral MTBC reference genome^55^ were generated with Gargammel v.1.1.2 1^98^ using four different length profiles (100 bp, 75 bp, 50 bp, and 30 bp) and damage patterns^99^. A mock oral community consisting of 20 bacterial taxa using identical Gargammel parameters was also generated. AdapterRemoval^90^ v2 trimmed, quality filtered, and merged paired-end reads. Each of the *M. tuberculosis* synthetic dataset were randomly sampled at 100,000, 50,000, and 10,000 reads using seqtk^100^ v.1.3 and spiked into randomly sampled mock oral community datasets so that each dataset consisted of a total of 10,000,000 reads, representing a relative abundance of 1%, 0.5%, and 0.1%, respectively. The simulated datasets were mapped against a reference database containing the ancestral MTBC reference genome^55^ and nine closely related *Mycobacterium* species to predict common sources of misidentification. Conda environment and all simulation scripts are available at https://github.com/aemann01/terry_tb_sim and are archived on Zenodo (10.5281/zenodo.4268790) for analytical reproducibility.

### Paleopathological assessment

Individuals were evaluated for pathological lesions, including trauma, and those associated with TB and syphilis/treponemal infection following established standards^30,31,62,65^. Diagnostic criteria for syphilis/treponemal infections and TB^1,32,65–67^ were synthesized and implemented as a single composite diagnostic score for each individual. This was done to cohesively estimate the probability of TB and syphilis infection (see Supplementary Table S1). Regardless of CoD, individuals were scored for skeletal lesions associated with both TB and syphilis/treponemal infection. Data from the osteological and paleopathological inventories were transcribed and coded in Excel. Analyses of frequency distributions of lesion types across anatomical regions and associations between composite diagnostic scores for TB and syphilis relative to the percentage of shotgun metagenomic reads mapped to the respective reference genome and high diagnostic value indicators for TB were conducted in R^93^.

### Ethical approval

Skeletal individuals used for this study are located at the Smithsonian Institution’s National Museum of Natural History within the Robert J. Terry Anatomical Collection. Due to on-going discussions concerning the largely unethical acquisition of human skeletal individuals for this collection prior to the mid-twentieth century, and opportunities for genealogical tracing for many individuals, we have chosen to de-identify individuals from their original Terry Collection accession numbers. While many individuals have associated names, the authors did not want to presume potential familial or community descendants would want to be connected to this scientific research, nor that the assessed individuals would want to be publicly remembered by name and in association with anatomical dissection or infectious diseases^74^. Original Terry Collection numbers are available upon request. Endogenous human DNA sequences have also been removed from the publicly available data to prevent individual and/or familial genetic identification, or identification of genetic life history measures (e.g., BRCA gene mutations).

### Supplementary Information

Supplementary Information 1.

Supplementary Information 2.

## Data Availability

Conda environment and all simulation scripts are available at https://github.com/aemann01/terry_tb_sim and are archived on Zenodo (10.5281/zenodo.4268790) for analytical reproducibility. The NGS sequencing data that support the findings of this study have been archived in NCBI Sequence Read Archive (SRA) under the BioProject PRJNA1056444. Human DNA sequences have been removed from the available SRA data to protect the genetic identity and potential genealogical descendants of the assessed individuals.
