# Publisher Correction: Metagenomic and paleopathological analyses of a historic documented collection explore ancient dental calculus as a diagnostic tool

**DOI:** 10.1038/s41598-024-69609-8

**Published:** 2024-08-12

**Authors:** Rita M. Austin, Tanvi P. Honap, Allison E. Mann, Alexander Hübner, Cassandra M. S. DeGaglia, Christina Warinner, Molly K. Zuckerman, Courtney A. Hofman

**Affiliations:** 1 Frontiers in Evolutionary Zoology Research Group, Natural History Museum of Oslo, University of Oslo, Oslo, 0562 Norway; 2 Department of Anthropology, National Museum of Natural History, Smithsonian Institution, Washington, DC 20560 USA; 3 Department of Anthropology, University of Oklahoma, Norman, OK 73019 USA; 4 Laboratories of Molecular Anthropology and Microbiome Research, University of Oklahoma, Norman, OK 73019 USA; 5 Department of Biological Sciences, Clemson University, Clemson, SC 29634 USA; 6 Department Archaeogenetics, Max-Planck-Institute for Evolutionary Anthropology, Leipzig, 04103 Germany; 7 Department of Anthropology, Tulane University, New Orleans, LA 70118 USA; 8 Department of Anthropology, Harvard University, Cambridge, MA 02138 USA; 9 Department of Anthropology and Middle Eastern Cultures, Mississippi State University, Mississippi State, MS 39762 USA

Correction to: *Scientific Reports* 10.1038/s41598-024-64818-7, published online 26 June 2024

The original version of this Article contained an error in Figure 2 where the cross-hatching and the shading is incorrect in panel A. The original Figure 2 and accompanying legend appear below.

**Figure 2:**
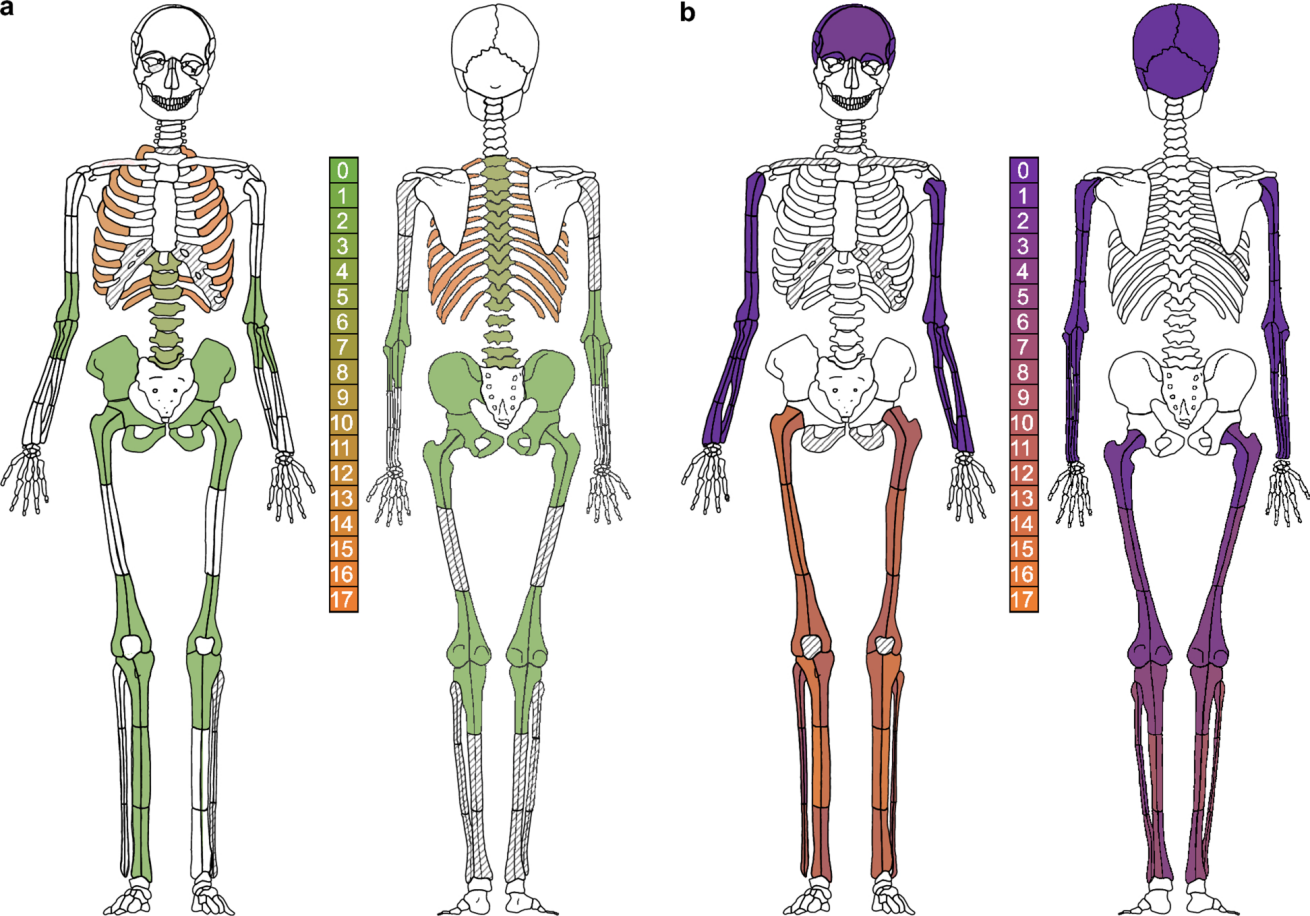
Frequency distributions of pathological skeletal lesions expressed as color gradients for skeletal elements across all 39 individuals, regardless of their recorded CoD. Frequency distributions (numbers) with color gradients (green and purple being less frequent than orange) of disease-indicating lesions are mapped onto an anatomical skeletal individual (modified from Buikstra and Ubelaker^62^). (**a**) Frequency distributions of lesions associated with TB (Supplementary Table S1, diagnostic criteria for TB); (**b**) frequency distributions of lesions associated with syphilis/treponemal infection (Supplementary Table S1, diagnostic criteria for syphilis). Cross hatching indicates skeletal elements that were not assessed for disease-indicating lesions.

The original Article has been corrected.

