# Supplementary material for: Metagenomic and paleopathological analyses of a historic documented collection explore ancient dental calculus as a diagnostic tool
Source: Sci Rep. 2024 Jun 26;14:14720. doi: 10.1038/s41598-024-64818-7 (PMC11208530; doi:10.1038/s41598-024-64818-7)
Supplement: Supplementary file 1 — Supplementary Information 1. [file 41598_2024_64818_MOESM1_ESM.pdf]

Supplementary Materials, Methods, and Figures

Title: Metagenomic and paleopathological analyses of a historic documented collection explore ancient dental calculus as a diagnostic tool

Authors: Rita M. Austin, Tanvi P. Honap, Allison E. Mann, Alexander Hübner, Cassandra M. S. DeGaglia, Christina Warinner, Molly K. Zuckerman, and Courtney A. Hofman

Materials and Methods

1. Terry Collection

1.1 Terry Collection Overview

The Terry Collection was assembled (1910-1967), cataloged, prepared, and stored under consistent and uniform protocols at the Washington University School of Medicine (WUSM) until its transfer to the Smithsonian Institution. The collection is known as one of the most complete and well documented anatomical collections in the world[1]. Information on individuals' chronological age, sex, social race, and cause of death (CoD) are known from associated documentation (e.g., death certificates). Individuals in this collection were never buried; upon being received at the WUSM, individuals underwent potent preparation, including manual maceration, boiling, drying, and degreasing by exposure to benzene vapors. This was followed by the application of a glue and formalin solution coating. For an in-depth account of the Terry Collection protocols and history see Hunt and Albanese[1], and Trotter[2].

1.2 People in the Terry Collection

Between 1910 and 1941 at the WUSM, Dr. Robert J. Terry amassed most of the individuals that would become the Robert J. Terry Collection. Prior to the mid-twentieth century, most of the individuals who were incorporated into the collection died in social relief institutions (e.g., asylums), after which they experienced non-consensual dissection (i.e., anatomization). Most of the individuals in the Terry are Black and/or African American (AA), many of whom were of low socioeconomic status (SES) to poor and arrived in Missouri via the Great Migration[3,4]. In the mid-twentieth century, Mildred Trotter PhD, who was Terry's colleague and a WUSM anatomy instructor, continued to expand the Terry until her 1967 retirement[4]. Trotter's efforts helped to balance the collection's sex demographics with the inclusion of more female individuals[1]. Due to socio-medical legislation, specifically the 1955 Missouri Willed Body Law, Trotter's collection methods also shifted towards incorporating more individuals who elected to donate their bodies. These individuals were typically of higher SES[2]. Even so, the Terry Collection persists as an example of the demographic biases seen across US historical documented collections, which are the direct result of 19th to mid-20th century processes of social and scientific structural and legislative violence against socially marginalized communities, both in life and after death[3–12]. Their skeletons embody the destructive effects of systemic poverty and race-based structural violence: many, especially the Black and/or AA individuals, have elevated rates of trauma, infectious disease, and markers of chronic physiological stress compared to the White and/or European Americans (EA) individuals[3–8,10,12]. Further, their dissection and incorporation into the Terry Collection (*vs*. burial) represents post-mortem structural violence, in part because anatomization was deeply stigmatized prior to the mid-twentieth century[3,6,10,12]. Individuals in the Terry did not consent to their inclusion within the collection nor any subsequent research, including this study, that was performed with the collection[3,6]. It is important to recognize this

history, as well as the complexity of these individuals' lives and remember them as posthumous contributors to this study (and so many others)[3,8]. At the same time, we also acknowledge that what constitutes ethical treatment of and research with human skeletal individuals varies cross culturally[10].

It is because of the relatively recent establishment of the Terry Collection (AD 1910-1967), the molecular revolution, and widespread use of genetic ancestry testing for personal and, to a growing extent, criminal investigations[9], that we use study-specific identifiers (*vs.* collection accession numbers) and removed reads mapping to the human genome from the analysis here and from files submitted to the NCBI Sequence Read Archive (http://www.ncbi.nlm.nih.gov/sra). These precautions were done in an effort to maintain the deceased individuals' genetic privacy, while also offering some posthumous bodily and individual respect and recognition[3]. Overall, these precautions are meant as mechanisms for recognizing these individuals as vital contributors to scientific work, rather than scientific objects[10,12]. Despite the Terry Collection's long established importance for theory and method-building within biological anthropology, clinical medicine, and related fields, these data precautions and ethical statements contribute to recent arguments that the Terry, like other historical documented collections, should no longer be considered or employed as neutral bodies of evidence within scientific contexts[3,5,8,10,12]. As part of this, we recognize that these precautions are not and should not be seen as a solution to recompense the complex histories that historical documented collections contain. Nor do they represent any form of practical or ethical solution to the social and scientific structural violence that these individuals endured antemortem and postmortem nor that much of the subsequent research upon them has perpetuated[3,4,6,8,10–12].

2. Molecular Analyses

2.1 Sampling

Subsampling of dental calculus was conducted at the National Museum of Natural History's (NMNH) Museum Support Center in the Biological Anthropology Collections, located in Suitland, Maryland. In accordance with previous studies[13], a bleach-sterilized dental scalar and nitrile gloves were used to scrape dental calculus directly from the individuals into a sterile 1.5 mL Eppendorf tube.

All laboratory work was performed at the Laboratories for Molecular Anthropology and Microbiome Research (LMAMR) at the University of Oklahoma in Norman, Oklahoma. Samples were processed in the LMAMR Ancient DNA Laboratory, a dedicated six-chambered clean room, following established guidelines for ancient DNA research. These include unidirectional workflow and positive air pressure to avoid cross-contamination. Researchers wore full-body Tyvek suits, masks, and gloves to prevent contamination of the samples. Non-template extraction and library negatives were processed in parallel to identify contamination introduced during the laboratory workflows.

2.2 DNA Extraction

DNA extraction was conducted following the protocol described in Ziesemer et al.[14] with slight modifications: no UV irradiation was applied to samples prior to the washing and digestion steps. Briefly, between 1 - 5.5 mg of dental calculus was washed with 0.5 M EDTA for 15 minutes before being allowed to decalcify in a solution of (fresh) 0.5 M EDTA and 10%

proteinase K (Qiagen) at room temperature on a nutator for 72 hours. Purification was done using a MinElute PCR Purification kit (Qiagen) with DNA being eluted in 60 µL Buffer EB. The extract was quantified using the High Sensitivity dsDNA assay on the Qubit fluorometer (Life Technologies).

2.3 Library Preparation

Prior to library construction, partial uracil-DNA-glycosylase (UDG) treatment was applied. This eliminates characteristic uracil damage from the interior of DNA molecules but preserves some of the terminal nucleotide damage. It thus allows for authentication of an ancient DNA signal[15]. 30 µL of DNA extract was used as input for partial UDG treatment. Following the partial UDG treatment and enzyme inactivation, samples were treated with the NEBNext DNA Library Prep Master Set (E6076, New England Biolabs) with slight modifications to the manufacturer's instructions. End-repair was completed by adding the NEBNext End Repair enzyme to the partial UDG reaction solution, followed by a 30-minute incubation at 20˚C. The end-repaired product was then purified using the MinElute PCR Purification kit (Qiagen) following the manufacturer's protocol and eluted in 15 µL Buffer EB. Illumina adapters were ligated using the NEBNext Quick Ligation Buffer and Quick T4 Ligase in a 15-minute incubation at 20˚C, followed by another MinElute purification with elution in 15 µL Buffer EB. An adapter fill-in was performed using the NEBNext Reaction Buffer and *Bst* Polymerase with incubation at 37˚C for 20 minutes, followed by a final MinElute PCR Purification kit (Qiagen) with elution in 50 µL Buffer EB. Libraries were quantified using a quantitative PCR (qPCR) assay using the KAPA HiFi Uracil+ enzyme to determine appropriate number of cycles for the indexing PCR, as given in Austin et al.[3].

Libraries were dual-indexed with two unique barcodes using the KAPA HiFi Uracil+ enzyme as given in Austin et al.[3]. Based on the qPCR cycle threshold values (Cq), libraries were split into three replicates, amplified to Cq + 4 number of cycles. Indexing PCR was performed as follows: initial denaturation at 98˚C for 5 minutes, followed by appropriate number of cycles of 98˚C (20s), 60˚C (15s), and 72˚C (30s), followed by a final elongation at 72˚C for 1 minute. PCR replicates for each sample were pooled and purified using a MinElute PCR Purification kit (Qiagen) with elution in 30 µL Buffer EB.

2.4 Shotgun Metagenomic Sequencing

Purified libraries were assessed using the Fragment Analyzer (Agilent, DNF-474 NGS) and pooled at equimolar ratios. The Pippin Prep (Sage Science) was used to remove adapter dimers by performing a size-selection at a target range of 150 - 500 bp. The resulting eluate was sequenced at The Max Planck Institute for the Science of Human History in Jena, Germany. It was sequenced on two Illumina NextSeq lanes using 1x75 bp or 2x150 bp read chemistry. Between 6.2 and 12 million reads were recovered, with extraction and library blanks sequenced to a depth of 0.4-8 million reads.

2.5 Data Filtering and Read Merging

Shotgun metagenomic data was processed using AdapterRemoval v2.1.7[16] to remove adapters and quality-filtered for base quality (q20) and read length (25 bp). Paired reads were merged with a minimum overlap of 11 bases. Quality-filtered, adapter trimmed, and, when applicable, merged (paired-end) reads are hereafter referred to as analysis-ready reads.

2.6 SourceTracker Analysis
SourceTracker v1.0.1[17] analysis was used to ascertain the contributions of taxa from known sources, such as oral, skin, soil samples, to the dental calculus samples from the Terry. Analysis-ready reads were mapped to the GreenGenes v13.08[18] database of bacterial and archaeal 16S rRNA gene sequences (97% pre-clustered) using Bowtie2[19] with default parameters and the --no-unal option. SAMTools v1.5[20] was used to sort the reads and remove PCR duplicates using *rmdup*. Unique reads mapping to the GreenGenes[18] database were extracted and used as input for closed-reference OTU-picking in QIIME v1.9.1[21] (uclust algorithm, 97% identity, max_accepts = 500, max_rejects = 500, word_length = 12, stepwords = 20, enable_rev_strand_match = True). The resulting BIOM table was rarefied to a depth of 4000 reads. A genus-level taxonomic summary was generated using QIIME[21] and used as input for SourceTracker[17] to assess the contribution of "source" samples such as human skin, supra- and subgingival plaque, calculus, and soil[22,23].

2.7 Reference-based mapping to T. p. pallidum, MTBC, and Common Oral Bacteria
A comparison of mapping rates among *T. p. pallidum*, MTBC, and common oral bacteria was done to assess the differential recovery and authentication of bacteria. Analysis-ready reads were mapped to genomes of *T. pallidum* (GCA 000604125.1), the ancestral MTBC reference genome[24], as well as *Fusobacterium nucleatum* (GCA 000007325.1) *Porphyromonas gingivalis* (GCA 000010505.1) and *Tannerella forsythia* (GCA 000238215.1) with Burrows-Wheeler Aligner (BWA) v0.7.12[25] with *aln* (seed length = 1000 and maximum edit distance = 0.1) and *samse* (default parameters). SAMTools[20] was used to sort the reads, filter unmapped (-F4) and low quality reads (minimum Phred score = 37), and remove PCR duplicates using *rmdup*.

2.8 Coverage Analysis
The three samples with the highest percentage of unique, quality filtered reads mapping to the ancestral MTBC reference genome[24] and *T. pallidum* (GCA 000604125.1) genomes were also visualized for the breadth and depth of read coverage using BRIG (BLAST Ring Image Generator)[26] with default BLASTN[27] parameters. Coverage was calculated across a 100bp window. Blue spikes in the coverage graphs indicate areas with read stacking in the genome (more than one standard deviation from the mean coverage) (Supplementary Figures S3 and S5).

2.9 HOPS Screening
The bioinformatics tool HOPS (Heuristic Operations for Pathogen Screening)[28] was used to screen the analysis-ready reads for presence of pathogenic species of interest. The HOPS reference database consisted of complete reference genomes of the species listed in Supplementary Figure S2, as available from NCBI Refseq (December 2016). Analysis-ready reads were then 'locally' aligned to bacterial genome references, particularly between highly conserved segments of the reference genomes.

2.10 MTBC Whole-Genome Capture
For bait preparation, genomic DNA from *M. tuberculosis* strains H37Rv (NR-48669), CDC1551 (NR-48981), East African Indian 91_0079 (NR-44095), and Indo-Oceanic T17X (NR-44096) was obtained through BEI Resources, NIAID, NIH, pooled in equal amounts (5 µg total), and used for generation of biotinylated RNA baits at Arbor Biosciences.

Two capture experiments, using two positive controls (Peruvian mummy samples 54U and 64U)[29] were conducted. An initial capture experiment was conducted to assess MTBC DNA recovery from Terry Collection individuals, ARP 1 T and ARP 9 T, and the positive control Peruvian sample 54U. High and low input DNA volumes for library preparation were tested with 15 µL input DNA for individuals' samples and 50 µL for the positive control. A second capture experiment was conducted to try to maximize detection of MTBC; when possible, new DNA extractions with higher extraction input weights, as well as larger library input volumes (40 µL DNA), were used for the selected individual's samples and the positive control sample 64U. For both capture experiments, each sample was enriched over two separate rounds of capture, as suggested by the manufacturer. 5.5 µL of biotinylated RNA baits were used in each round of capture. Hybridization was conducted at 65ºC for 12 hours, immediately followed by 60˚C for 36 hours.

After each round of capture, enriched libraries were amplified following manufacturer's protocol and using the KAPA HiFi HotStart kit (KAPA Biosystems), with 15 µL of capture library being amplified over three replicates (5 µL per replicate). Amplified libraries were pooled and purified using an AMPure bead cleanup to remove fragments shorter than 150 bp and eluted in 30 µL of Buffer EB (Qiagen). Enriched libraries were quantified using a Fragment Analyzer (Agilent) and pooled in equimolar ratios. This was followed by size-selection using the PippinPrep system (Sage Science) at a target size range of 150-500 bp, prior to sequencing on an Illumina NextSeq 2x150 bp run.

2.11 Capture Data Analysis

Adapter removal and read merging mirrored the parameters used for shotgun metagenomic data (see section 2.5 above). Analysis-ready reads for 54U, 64U, ARP 1 T, and ARP 9 T were mapped to the ancestral MTBC reference genome[24], using BWA[25]. Post-mapping quality-filtering was done using SAMTools[20], respectively, following the same parameters listed in section 2.7 above. To authenticate the origins of these mapped reads, they were used as queries in a BLASTN[27] search (e-value = 1e-6, max_target_seqs = 5) against the complete NCBI-nt database. Sequence data from each capture experiment was combined for ARP 1 T and ARP 9 T prior to the BLASTN[27] search. Reads were removed using FilterSamReads (Picard tools v2.20.3[30]) if any of the top five matches was not MTBC. Variant calling was conducted on the filtered BAM files using SAMTools[20] *mpileup* (include all positions = -a) and VarScan2[31] *mpileup2cns* (minimum read depth = 5, minimum supporting reads = 3, minimum base quality = 37, minimum variant allele frequency threshold = 0.2, minimum frequency to call homozygote = 0.9, p-value threshold = 1, strand filter = 0). Damage patterns were assessed using MapDamage v2.2.1[32]. MTBC lineages were determined with the SNP-IT tool[33].

3. Skeletal Analyses

Following established standards, individuals (n=39) were inventoried for pathological conditions, including trauma, and skeletal indicators of overall health[34–37], as well as skeletal manifestations associated with syphilis/ treponemal infection and TB[35,36,38,39]. Diagnostic criteria based on several previous studies[35,36,38–40] were synthesized and implemented in order to cohesively assess skeletal lesions associated with TB infection (see Supplementary Table S1). A composite score of pathologic lesions for TB[38,40,41] and syphilis/ treponemal infection[39] was

determined for each individual as a diagnostic estimate of the probability of infection with syphilis and/or TB. Regardless of CoD, individuals were evaluated and scored for both TB and syphilis/ treponemal infection.

Supplementary Figures

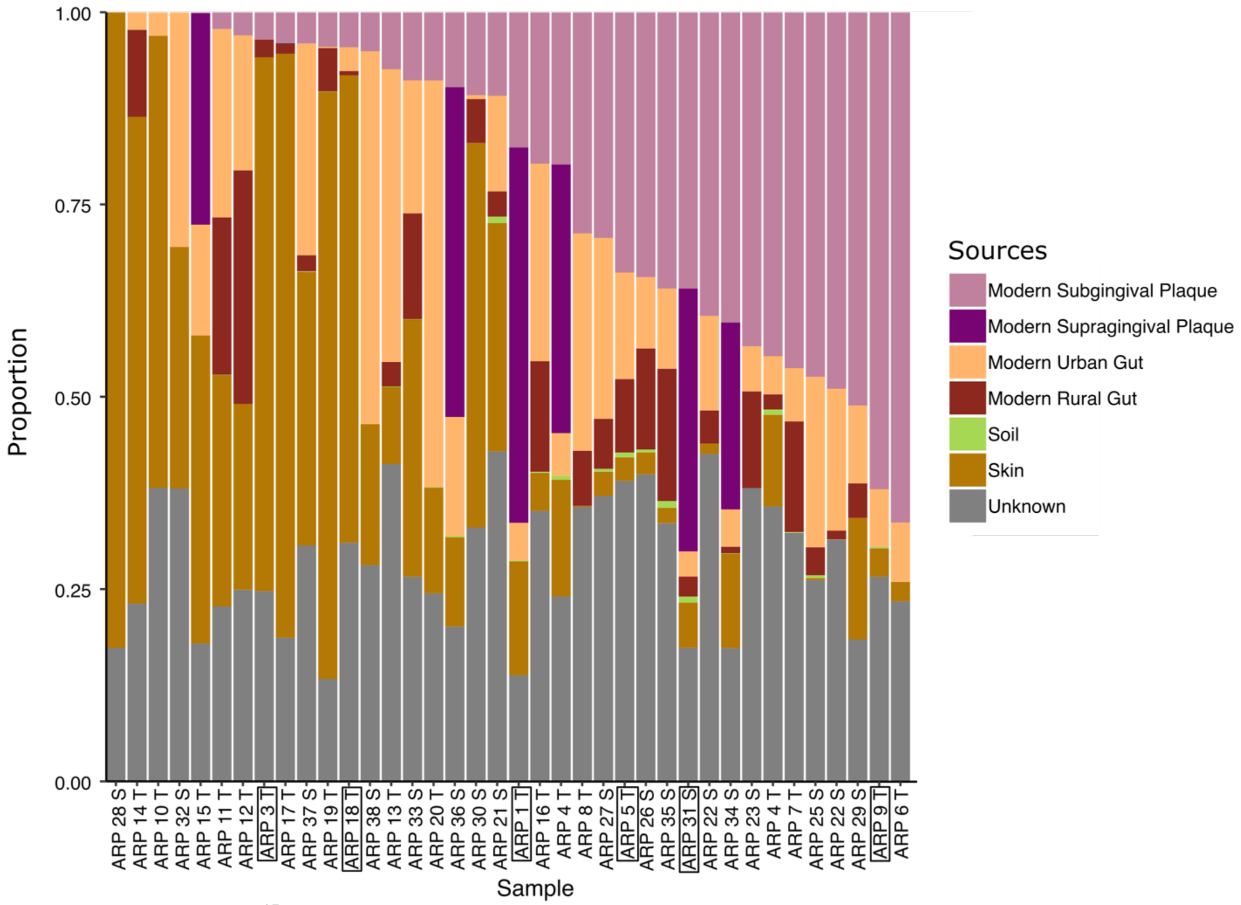

Figure S1. SourceTracker[17] analysis of 16S rRNA metagenomic reads sorted by increasing proportions of taxa associated with modern subgingival plaque. Boxed and bolded sample names indicate samples used in hybridization capture experiments. In only using modern community profiles for comparison samples show lower proportions of taxa related to oral communities.

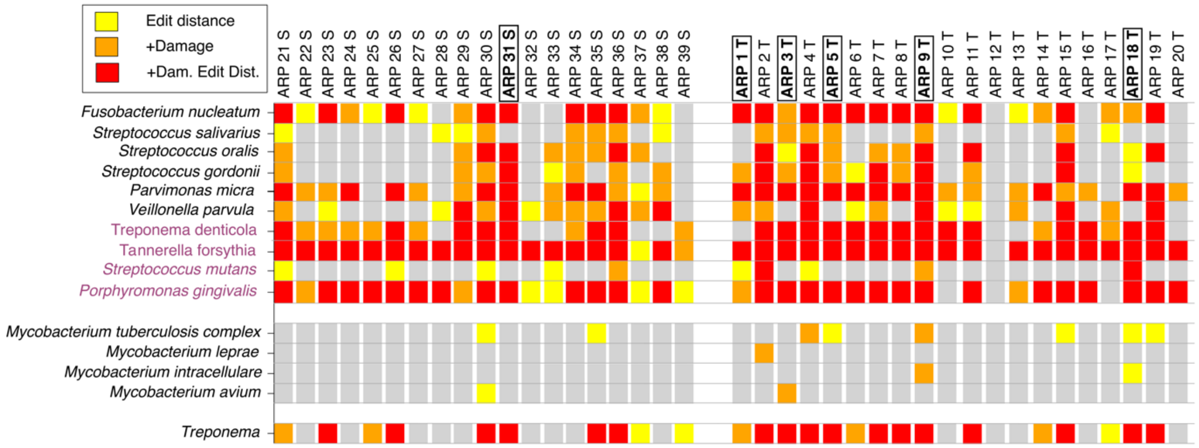

Figure S2. Heatmap of HOPS[28] identification results from dental calculus. Color indicates surety of detection, with red having both, detectable damage patterns and edit distance. Bolded and boxed samples were subjected to *M. tuberculosis* hybridization capture. Purple font species are common oral pathogens. *Mycobacterium* species were not detected across samples. When screening for various *Treponema* species, only *T. putidum* and *T. denticola* were detected across samples; *T. pallidum* was not detected.

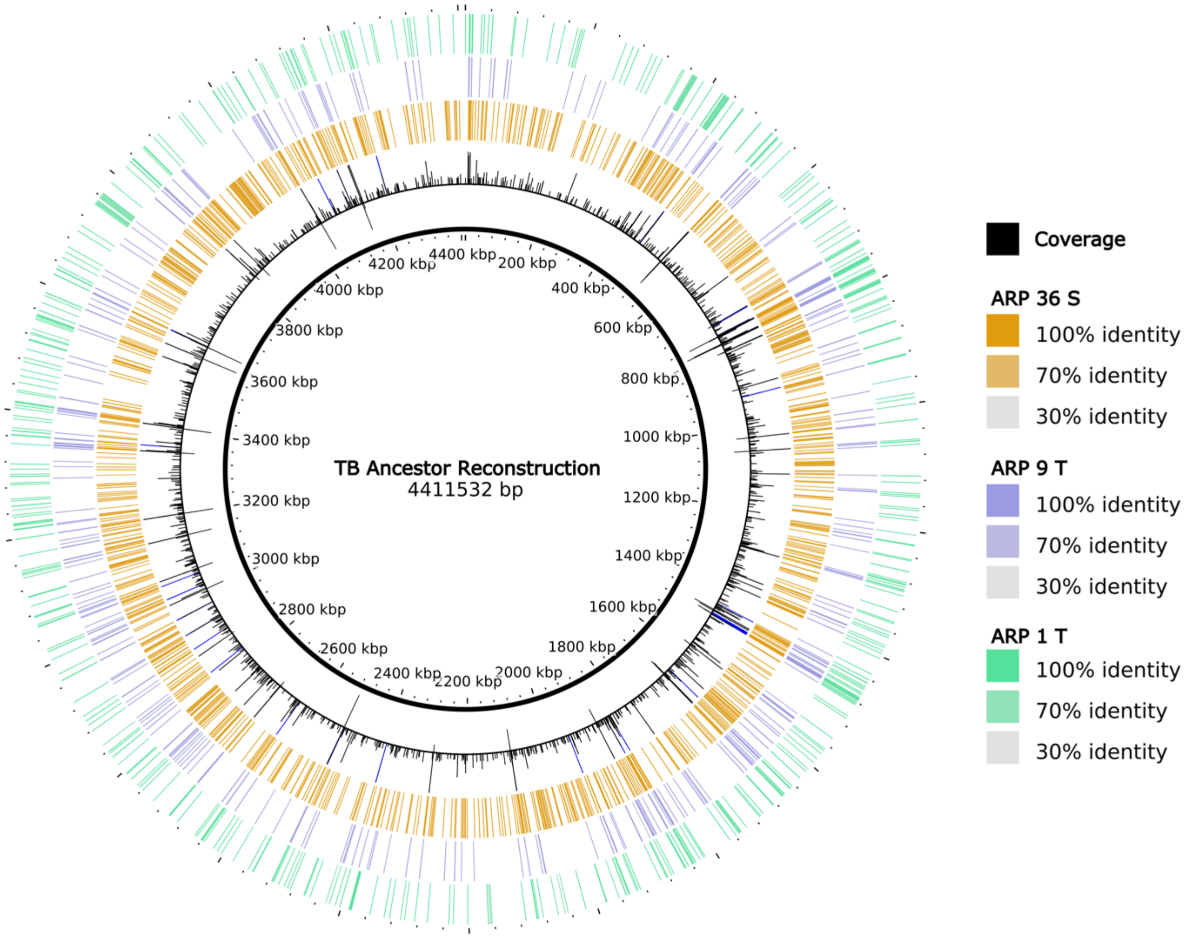

Figure S3. Genome coverage and percent identity of the top three samples (ARP 36 S, ARP 1 T, and ARP 9 T) with unique reads mapping to the *M. tuberculosis* reconstructed genome[24]. Generated using the BRIG[26], tick marks indicate reads and their position on the reference genome (colored outer rings) with the percent identity (shades of colored rings) indicating read similarity to the reference genome as determined in BLAST results. The innermost, black ring represents the reference genome while the secondary black ring represents genome coverage of all three samples merged together, calculated as the average over a rolling base-pair window (100bp), at particular locations of the reconstructed *M. tuberculosis* genome. A large portion of the reference genome was covered with high percent identities (dark orange, purple, and green ticks).

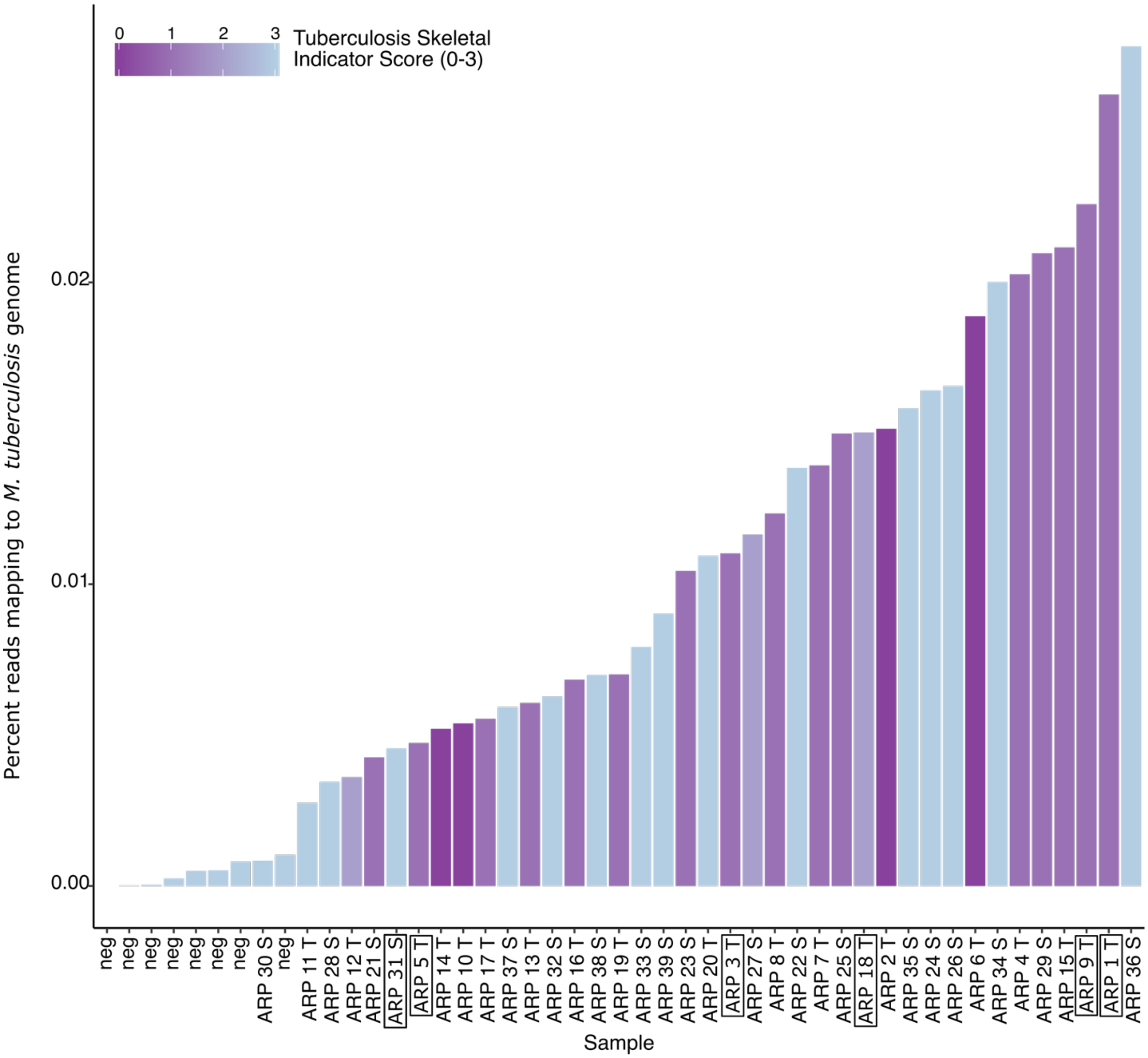

Figure S4. Percent of shotgun metagenomic reads mapping to *M. tuberculosis* genome reconstruction[24] by sample, colored by scored TB skeletal diagnostic indicator score. Bolded and Boxed samples indicate those selected for *M. tuberculosis* hybridization capture. There is not an apparent relationship between skeletal indicator scores and the percentage of sequencing reads mapping to *M. tuberculosis*[24] from the assessed dental calculus.

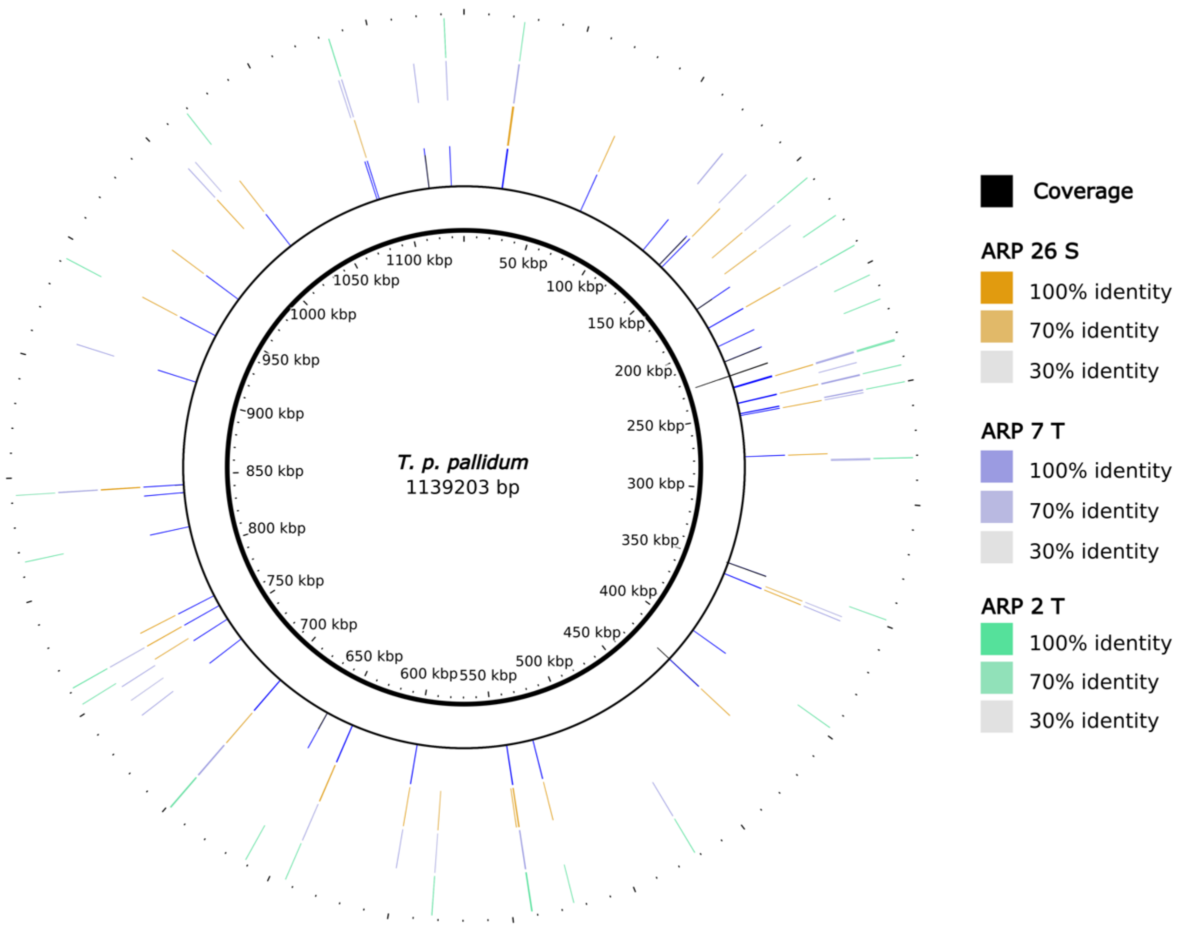

Figure S5. Genome coverage and percent identity of the top three samples (ARP 7 T, ARP 2 T, and ARP 26 S) with unique reads mapping to *T. pallidum* (*T. pallidum* subsp. *pallidum* str. Sea 81-4). Generated using the BRIG[26], tick marks indicate reads and their position on the reference genome (three outermost colored rings) with the percent identity (shades of colored rings) indicating read similarity to the reference genome as determined in BLAST results. The innermost, black ring represents the reference genome while the secondary inner black ring represents genome coverage of all three samples merged together, calculated as the average over a rolling base-pair window (100bp), at particular locations of the *T. pallidum* genome. Genome regions with coverage more than one standard deviation from the mean coverage are represented as blue spikes. While reads were recovered across the *T. pallidum* genome, only a very small proportion of the total genome was retrieved. Additionally, high read stacking (blue ticks in coverage plot) is seen across samples.

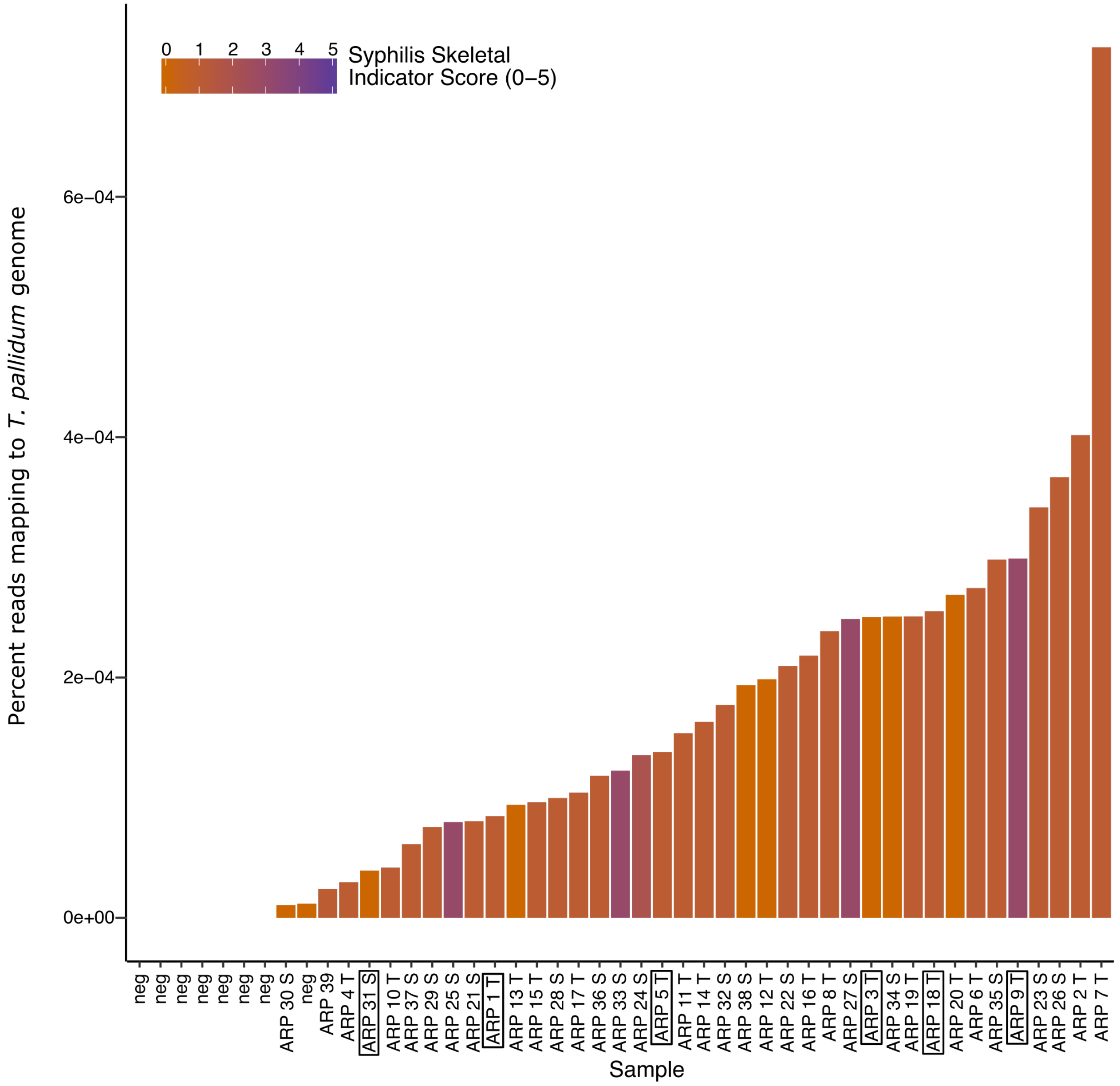

Figure S6. Percent of shotgun metagenomic reads mapping to *T. pallidum* (str. Sea 81-4) by sample. Boxed and bolded sample names indicate samples selected for *M. tuberculosis* hybridization capture. There is not an apparent relationship between skeletal indicator scores and the percentage of sequencing reads mapping to *T. pallidum* (str. Sea 81-4) from the assessed dental calculus.

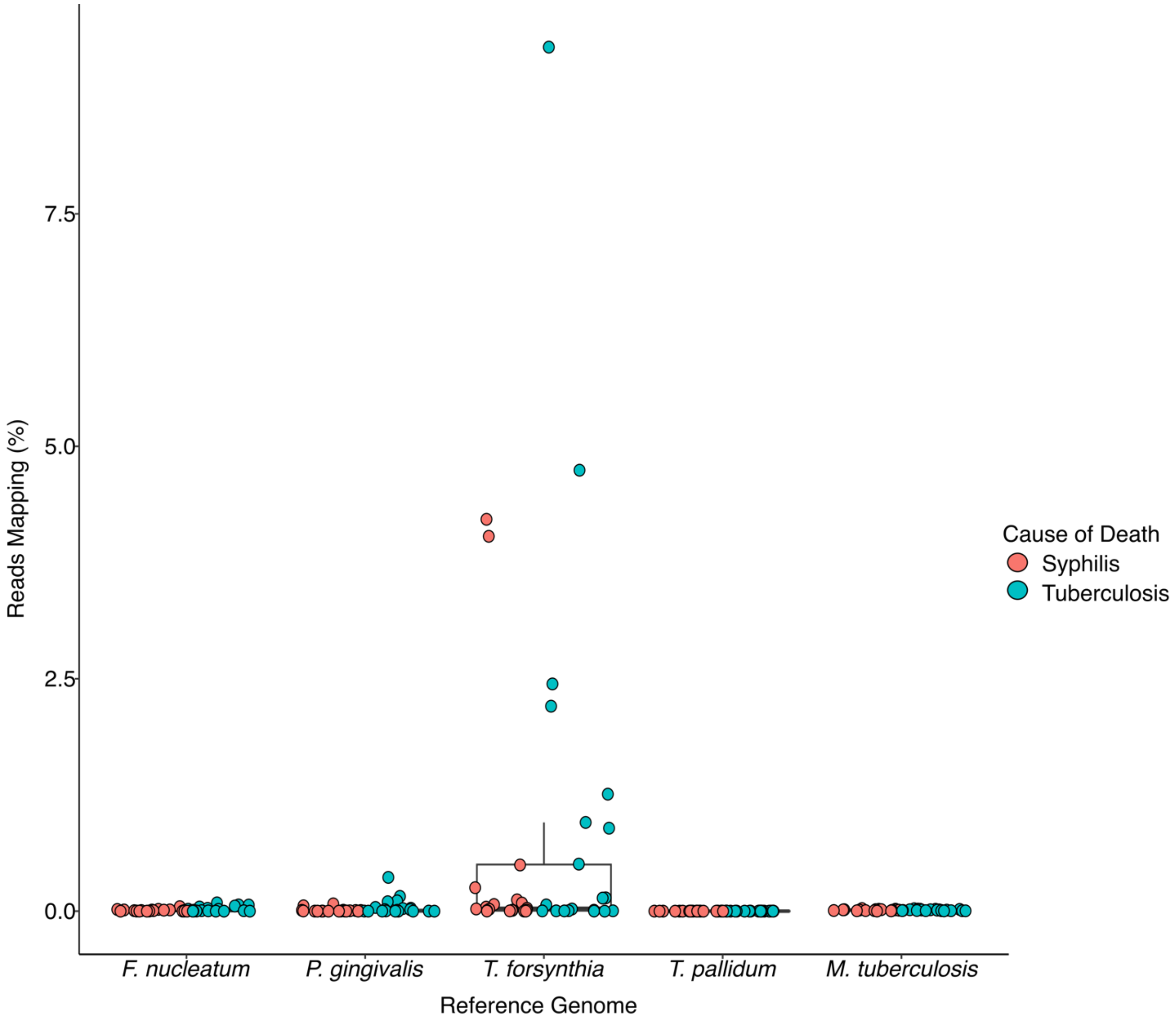

Figure S7. Proportion of unique, quality filtered reads mapped to genomes of *F. nucleatum* (GCA 000007325.1), *P. gingivalis* (GCA 000010505.1), *T. forsynthia* (GCA 000238215.1), *T. pallidum* (GCA 000604125.1), and *M. tuberculosis*[24].

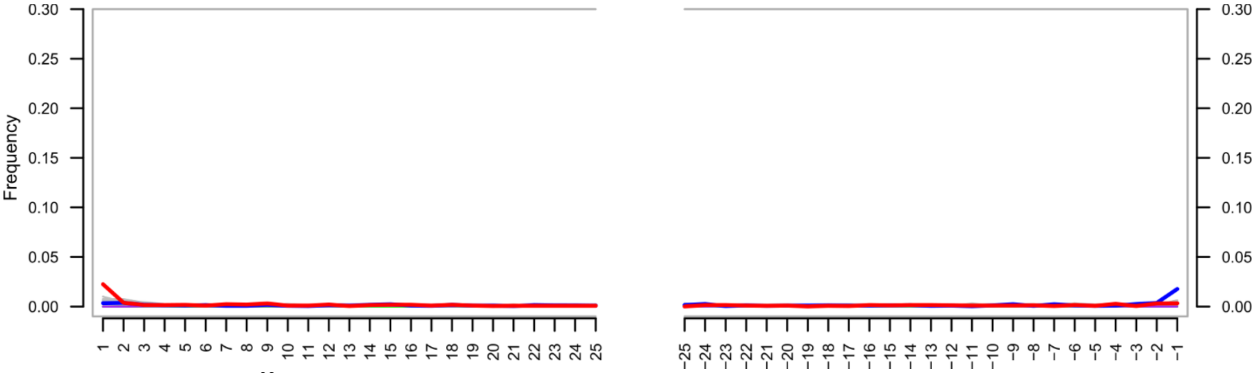

Figure S8. MapDamage[32] plot for ARP 1 T MTBC-genome capture data mapped to the *M. tuberculosis* ancestral reference genome[24], after BLASTN[27] filtering.

<u>Supplementary Materials References</u>

1. Hunt, D. R. & Albanese, J. History and demographic composition of the Robert J. Terry anatomical collection. *Am J Phys Anthropol* **127**, 406–417 (2005).
2. Trotter, M. Robert J. Terry, 1871–1966. *Am J Phys Anthropol* **56**, 503–508 (1981).
3. Austin, R. M. *et al.* Remembering St. Louis individual—structural violence and acute bacterial infections in a historical anatomical collection. *Communications Biology* **5**, 1–10 (2022).
4. de la Cova, C. Marginalized bodies and the construction of the Robert J. Terry anatomical skeletal collection: A promised land lost. in *Bioarchaeology of marginalized people* 133–155 (Elsevier, 2019).
5. Muller, J. L., Pearlstein, K. E. & de la Cova, C. Dissection and documented skeletal collections: embodiments of legalized inequality. in *The bioarchaeology of dissection and autopsy in the United States* 185–201 (Springer, 2017).
6. Zuckerman, M.K., Austin, R.M., & Hofman, C.A. Historical anatomical collections of human remains: Exploring their reinterpretation as representations of racial violence. *Ann Am Acad Polit SS* **694**, 39–47 (2021).
7. de la Cova, C. Race, health, and disease in 19th-century-born males. *Am J Phys Anthropol* **144**, 526–537 (2011).
8. Mant, M., de la Cova, C. & Brickley, M. B. Intersectionality and trauma analysis in bioarchaeology. *Am J Phys Anthropol* **174**, 583–594 (2021).
9. Gafni, M. & Krieger, L. Here's the 'open-source'genealogy DNA website that helped crack the Golden State Killer case. *Mercury News* **26**, (2018).
10. de la Cova, C. Ethical Considerations for Paleopathology. in *The Routledge Handbook of Paleopathology* 381–396 (Routledge, 2022).
11. Watkins, R. Anatomical collections as the anthropological other: Some considerations. in *Bioarchaeological Analyses and Bodies* 27–47 (Springer, 2018).
12. de la Cova, C. Making silenced voices speak. in *Theoretical Approaches in Bioarchaeology* (Routledge, 2020).
13. Warinner, C. *et al.* Pathogens and host immunity in the ancient human oral cavity. *Nat Genet* **46**, 336–344 (2014).
14. Ziesemer, K. A. *et al.* Intrinsic challenges in ancient microbiome reconstruction using 16S rRNA gene amplification. *Sci Rep* **5**, 16498 (2015).

15. Rohland, N., Harney, E., Mallick, S., Nordenfelt, S. & Reich, D. Partial uracil–DNA–glycosylase treatment for screening of ancient DNA. *Phil Trans R Soc B* **370**, (2015).
16. Schubert, M., Lindgreen, S. & Orlando, L. AdapterRemoval v2: Rapid adapter trimming, identification, and read merging. *BMC Res Notes* **9**, 1–7 (2016).
17. Knights, D. *et al.* Bayesian community-wide culture-independent microbial source tracking. *Nat Methods* **8**, 761–763 (2011).
18. DeSantis, T. Z. *et al.* Greengenes, a chimera-checked 16S rRNA gene database and workbench compatible with ARB. *Appl Environ Microbiol* **72**, 5069–5072 (2006).
19. Langmead, B. & Salzberg, S. L. Fast gapped-read alignment with Bowtie 2. *Nat Methods* **9**, 357 (2012).
20. Li, H. *et al.* The sequence alignment/map format and SAMtools. *Bioinformat* **25**, 2078–2079 (2009).
21. Caporaso, J. G. *et al.* QIIME allows analysis of high-throughput community sequencing data. *Nat Methods* **7**, 335 (2010).
22. Mann, A. E. *et al.* Differential preservation of endogenous human and microbial DNA in dental calculus and dentin. *Sci Rep* **8**, (2018).
23. Velsko, I. M. *et al.* Microbial differences between dental plaque and historic dental calculus are related to oral biofilm maturation stage. *Microbiome* **7**, 1–20 (2019).
24. Comas, I. *et al.* Human T cell epitopes of Mycobacterium tuberculosis are evolutionarily hyperconserved. *Nat Genet* **42**, 498 (2010).
25. Li, H. & Durbin, R. Fast and accurate short read alignment with Burrows–Wheeler transform. *Bioinformat* **25**, 1754–1760 (2009).
26. Alikhan, N.-F., Petty, N. K., Zakour, N. L. B. & Beatson, S. A. BLAST Ring Image Generator (BRIG): simple prokaryote genome comparisons. *BMC genomics* **12**, 402 (2011).
27. Altschul, S. F., Gish, W., Miller, W., Myers, E. W. & Lipman, D. J. Basic local alignment search tool. *Journal of molecular biology* **215**, 403–410 (1990).
28. Hübler, R. *et al.* HOPS: Automated detection and authentication of pathogen DNA in archaeological remains. *Genome biology* **20**, 1–13 (2019).
29. Bos, K. I. *et al.* Pre-Columbian mycobacterial genomes reveal seals as a source of New World human tuberculosis. *Nature* **514**, 494–497 (2014).
30. Broad Institute. Picard toolkit. *Broad Institute, GitHub repository* (2019).
31. Koboldt, D. C. *et al.* VarScan 2: Somatic mutation and copy number alteration discovery in cancer by exome sequencing. *Genome research* **22**, 568–576 (2012).
32. Jónsson, H., Ginolhac, A., Schubert, M., Johnson, P. L. & Orlando, L. mapDamage2. 0: Fast approximate Bayesian estimates of ancient DNA damage parameters. *Bioinformat* **29**, 1682–1684 (2013).
33. Lipworth, S. *et al.* SNP-IT tool for identifying subspecies and associated lineages of Mycobacterium tuberculosis complex. *Emerging infectious diseases* **25**, 482 (2019).
34. Buikstra, J. E. & Ubelaker, D. H. Standards for recording human remains. *Ark Archaeol Sur Res* (1994).
35. Ortner, D. J. *Identification of Pathological Conditions in Human Skeletal Remains*. (Academic Press, 2003).
36. Buikstra, J. E. *Ortner's Identification of Pathological Conditions in Human Skeletal Remains*. (Academic Press, 2019).
37. Powers, N. Human osteology method statement. *London: Museum of London* (2012).

38. Dangvard Pedersen, D., Milner, G. R., Kolmos, H. J. & Boldsen, J. L. The association between skeletal lesions and tuberculosis diagnosis using a probabilistic approach. *Int J Paleopathol* **27**, 88–100 (2019).
39. Harper, K. N., Zuckerman, M. K., Harper, M. L., Kingston, J. D. & Armelagos, G. J. The origin and antiquity of syphilis revisited: An appraisal of Old World pre-Columbian evidence for treponemal infection. *American Journal of Physical Anthropology* **146**, 99–133 (2011).
40. Santos, A. L. & Roberts, C. A. Anatomy of a serial killer: differential diagnosis of tuberculosis based on rib lesions of adult individuals from the Coimbra Identified Skeletal Collection, Portugal. *American Journal of Physical Anthropology: The Official Publication of the American Association of Physical Anthropologists* **130**, 38–49 (2006).
41. Steckel, R. H., Larsen, C. S., Sciulli, P. W., Walker, P. L., & others. Data collection codebook. *The global history of health project* **2006**, 1–41 (2006).
